# Treatment combining RU486 and Ad5IL-12 vector attenuates the growth of experimentally formed prostate tumors and induces changes in the sentinel lymph nodes of mice

**DOI:** 10.1186/1479-5876-8-98

**Published:** 2010-10-14

**Authors:** Claudia Raja Gabaglia, Alexandra DeLaney, Jennifer Gee, Ramesh Halder, Frank L Graham, Jack Gauldie, Eli E Sercarz, Todd A Braciak

**Affiliations:** 1 Division of Immune Regulation, Torrey Pines Institute for Molecular Studies (TPIMS), 3550 General Atomics Court, San Diego, CA 92121, USA; 2 Laboratory of Autoimmunity, Torrey Pines Institute for Molecular Studies (TPIMS), 3550 General Atomics Court, San Diego, CA 92121, USA; 3 Department of Pathology and Molecular Medicine, McMaster University, 1200 Main Street West, Hamilton, ONT, L8N 3Z5, Canada

## Abstract

**Background:**

Tumor immune responses are first generated and metastases often begin in tumor sentinel lymph nodes (TSLN). Therefore, it is important to promote tumor immunity within this microenvironment. Mifepristone (RU486) treatment can interfere with cortisol signaling that can lead to suppression of tumor immunity. Here, we assessed whether treatment with RU486 in conjunction with an intratumor injection of Ad5IL-12 vector (a recombinant adenovirus expressing IL-12) could impact the TSLN microenvironment and prostate cancer progression.

**Methods:**

The human PC3, LNCaP or murine TRAMP-C1 prostate cancer cell lines were used to generate subcutaneous tumors in NOD.*scid *and C57BL/6 mice, respectively. Adjuvant effects of RU486 were looked for in combination therapy with intratumor injections (IT) of Ad5IL-12 vector in comparison to PBS, DL70-3 vector, DL70-3 + RU486, RU486 and Ad5IL-12 vector treatment controls. Changes in tumor growth, cell cytotoxic activity and populations of CD4^+^/FoxP3^+ ^T regulatory cells (Treg) in the TSLN were evaluated.

**Results:**

Treatment of human PC3 prostate xenograft or TRAMP-C1 tumors with combination Ad5IL-12 vector and RU486 produced significantly better therapeutic efficacy in comparison to controls. In addition, we found that combination therapy increased the capacity of TSLN lymphocytes to produce Granzyme B in response to tumor cell targets. Finally, combination therapy tended towards decreases of CD4^+^/FoxP3^+ ^T regulatory cell populations to be found in the TSLN.

**Conclusion:**

Inclusion of RU486 may serve as a useful adjuvant when combined with proinflammatory tumor killing agents by enhancement of the immune response and alteration of the TSLN microenvironment.

## Background

Prostate cancer is one of the leading causes of death in men and has not been curable once it has metastasized beyond the local prostate gland [1]. This poor effect of current therapy on metastases could be the result of immunosuppressive conditions found in tissue microenvironments where metastatic cancer cells migrate including the TSLN. The TSLN is defined as the lymph node to first receive lymphatic drainage from the primary tumor site and is the first lymphoid organ that can respond to tumor challenge [2]. In patients, the status of the TSLN is one of the most significant predictors of overall survival for most clinical stage I/II solid tumors [3,4]. An immune phenotype in which suppressive cytokines are predominantly produced by Treg cells amongst TSLN cells is usually associated with failure to prevent tumor metastases [5]. Importantly with regard to various immune-therapeutic interventions, Treg populations have been shown to possess a capacity for plasticity and can be converted from a suppressive to activated phenotype given the appropriate stimulation [6,7]. Therefore, novel therapies that override TSLN immunosuppression may restore effective tumor immunity.

We have previously used a recombinant adenovirus vector expressing the IL-12 cytokine (Ad5IL-12) in combination with mitotane, a drug that transiently suppresses cortisol production, to enhance the activity of the vector and produce more successful therapy of experimental prostate cancers in mice [8]. Cortisol can act on lymphocytes and dendritic cells (DC) to suppress the expression of proinflammatory cytokines and costimulatory molecules, factors that have been shown to be important for the generation of immune responses against tumors [9]. This study indicated that cortisol can contribute to defects in immune function that allow tumor escape. Because mitotane has an associated toxicity when used in treatment, we decided to test the effects of cortisol receptor blockade using the drug mifepristone (RU486). Mifepristone is a progesterone analogue that can act as an antagonist for the glucocorticoid receptor (GR) [10]. Therefore, we examined RU486 treatment in combination with the Ad5IL-12 vector to determine if this combination could similarly influence (as mitotane treatment) prostate cancer progression. Therapies incorporating combinations of adenovirus vectors with various immune stimulatory agents have been shown to produce better therapeutic outcomes [11-13]. Given that RU486 is an approved pharmaceutical and affect pathways of homeostatic regulation, we sought to evaluate whether it would also be useful as an immunological adjuvant in cancer therapy.

Factors that influence the tissue microenvironment of the TSLN include the production of immunosuppressive cytokines. One of the most important suppressive cytokines controlling immune response is IL-10. IL-10 has been shown to generally suppress T cell immune responses and elevated levels of this cytokine have been detected in the serum of prostate cancer patients compared to normal healthy controls [14]. Tumor infiltrating lymphocytes isolated from prostate cancers have significantly higher IL-10 expression than T lymphocytes from peripheral blood, indicating IL-10 can influence cells in the tumor microenvironment and immune response [15]. Another prominent inhibitory cytokine, transforming growth factor-beta (TGF-β) can be produced by prostate cancer cells and has been shown to inhibit prostate tumor immunity [16]. TGF-β has a negative impact on immune function where it has been shown to suppress T cell activation and chemotaxis, as well as to inhibit DC maturation and function [17]. Additionally, studies have demonstrated an inverse correlation to survival when higher levels of TGF-β are detected in the serum or produced by tumor cells isolated from prostate cancer patients [18,19].

Importantly, cortisol can induce the production of both suppressive cytokines (IL-10 and TGF-β) and could orchestrate hormonal control upon immune response within the TSLN microenvironment. In association to human studies, a dysregulated diurnal cortisol cycle was found to correspond to lower 5 year survival outcomes for breast cancer patients, supporting an importance of sustained cortisol levels to poorer clinical outcomes [20]. In addition as cortisol can control the production of IL-10 and TGF-β, these cytokines have been linked to the establishment of immune suppression in the tumor microenvironment by aiding in the expansion of FoxP3^+ ^regulatory T cells (Treg) [21-23]. Treg cells have been shown to negatively affect tumor immunity as the depletion of CD4^+^CD25^+^FoxP3^+ ^Treg from tumor tissue and the TSLN has been shown to facilitate tumor rejection [24-26]. Therefore, it is possible that therapies affecting cortisol response could downregulate Treg activity in the TSLN and aid in the generation of effective tumor immunity.

In this report, we demonstrate experimental prostate tumors benefit from the inclusion of RU486 treatments in combination with IT injection of Ad5IL-12 vector. We find that this combination therapy has a greater attenuating effect on the growth of both human androgen-independent PC3 xenograft tumors in NOD.*scid *mice as well as TRAMP-C1 tumors formed in C57BL/6 mice. With the addition of mifepristone treatment to the Ad5IL-12 vector, cytotoxic activity in the TSLN is enhanced. These results indicate that the inclusion of RU486 in a proinflammatory-based prostate cancer immunotherapy can favorably alter the TSLN microenvironment to improve treatment efficacy.

## Materials and methods

### Mice and tumor cell lines

Six- to eight-week old male NOD.*scid *and C57BL/6 mice were obtained from the Jackson Laboratory (Bar Harbor, MD) and bred in the animal facilities at TPIMS. All work was done according to TPIMS guidelines for animal use and care. The TPIMS Institutional Animal Care and Use Committee provided approval (TPI-08-02) that covers the ethical use of animals in experimentation and all experimental research on animals followed internationally recognized guidelines. The human prostate cancer cell line PC3 was grown in Dulbecco's modified Eagle's medium (DMEM), supplemented with 10% fetal bovine serum (FBS), 100 μg/ml streptomycin and 100 IU/ml of penicillin. The androgen-dependent LNCaP cells were additionally supplemented with 10^-8 ^M dihydrotestosterone. TRAMP-C1 tumor cells were passaged serially without dihydrotestosterone to establish androgen-independent growth for use in this study. All cell lines were obtained from American Type Culture Collection (Manassas, VA).

### Establishment of tumor and treatment protocol

The human PC3, LNCaP or murine TRAMP-C1 prostate cancer cell lines were used to generate subcutaneous tumors in NOD.*scid *and C57BL/6 mice. Two million tumor cells in 50 μl of PBS were mixed with 50 μl of matrigel and injected subcutaneously (SC) in the right hind flank of animals. Intratumor injections (IT) were given with a 5 × 10^8 ^pfu dose of adenovirus vectors in 50 μl volumes of PBS using a 26-gauge needle when palpable tumors formed (approximately 3 weeks). Tumor growth was monitored weekly by measurment in two dimensions using a caliper and volumes calculated assuming a prolate spheroid tumor mass as previously described [27]. Mifepristone/RU486 [17β-hydroxy-11β-(4-dimethylaminophenyl)-17α-1-propyl-estra-4,9-dien-3-one] catalog M8046 was purchased from Sigma-Aldrich (St. Louis, MO). For use in intraperitoneal administrations (IP), 200 μl volumes of microcrystalline RU486 (25 μg/g of weight) were freshly prepared in sterile PBS as previously described [28].

### Adenovirus vectors

The construction of the Ad5IL-12 and the DL70-3 adenovirus type 5 vectors (Ad5) used in this study are previously described [27]. The Ad5IL-12 vector is a replication incompetent recombinant adenovirus type 5 (Ad5) that encodes the p35 subunit of IL-12 in the E1 region and the p40 subunit in the E3 region of the Ad5 virus genome. The DL70-3 control Ad5 vector is a replication incompetent adenovirus depleted of E1 region sequences and expresses no transgene. All vectors used in this study were propagated in 293 cells and purified on cesium chloride gradients as previously described [29].

### TSLN Granzyme B measurement

The mouse granzyme B ELISA kit used to measure granzyme B production from isolated TSLN lymphocytes was supplied by eBIOSCIENCE (San Diego, CA). TSLN cells were prepared from individual mice bearing TRAMP-C1 tumors from each treatment group (PBS, RU486, DL70-3, Ad5IL-12 and Ad5IL-12 + RU486) at the end of 7 days (the endpoint of RU486 therapy) and incubated for 24 hrs with irradiated TRAMP-C1 cells as targets. 1 × 10^6 ^TRAMP-C1 irradiated target cells (3000 r cumulative dose) were cultured alone or co-cultured with 1 × 10^6 ^TSLN cells at 37°C in 24-well tissue culture plates in a volume of 500 μl of complete DMEM media. At the end of this incubation period, supernatants were collected and analyzed for granzyme B content as per the manufacturer's instructions.

### Flow Cytometry

Characterization by flow cytometry analysis of cell surface expression of Ly49C and CD4 on TSLN lymphocytes was performed with FITC-labeled anti-Ly49C and anti-CD4 mAbs. For CD25 detection, an APC-labeled anti-CD25 mAb was used. For intracellular detection, a PE-labeled anti-FoxP3 mAb was used. All antibodies and isotype controls were purchased from BD Biosciences (San Diego, CA). All analysis was performed on a FACSCalibur flow cytometer (Becton Dickinson, Mountain View, CA).

### Statistics

Statistical analysis was performed using the STATVIEW 4.5 program from Abacus Concepts (Berkeley, CA) by Student's *t*-test for final determination of significance.

## Results

### RU486 augments antitumor activity of Ad5IL-12 in PC3 xenograft model

**Figure 1:**
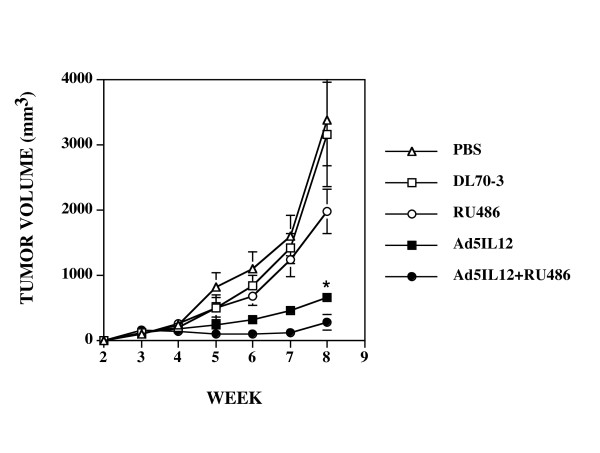
**Intratumoral injection with Ad5IL-12 vector and 1 week treatment with RU486 synergistically attenuates the growth of human PC3 tumors**. Xenograft tumors established SC in NOD.*scid *mice were treated at week 3 by IT injection with 50 μl of PBS containing 5 × 10^8 ^pfu of Ad5IL-12 (filled squares) or control DL70-3 vector (empty squares) or PBS alone (empty circles). In addition, another set of mice were treated with Ad5IL-12 IT injection and given daily IP injections of RU486 for 7 days (black triangles). Data points are expressed as the mean ± SE. n = 8 for each data point. * indicates statistical significance of P < 0.05 for Ad5IL-12 + RU486 treatments alone compared to controls. Tumor volumes measured at 8 weeks were 3394 ± 87 mm^3 ^for PBS, 3163 ± 87 mm^3 ^for DL70-3, 1989 ± 307 for RU486, 668 ± 87 mm^3 ^for Ad5IL-12 and 298 ± 120 mm^3 ^for Ad5IL-12 + RU486 treatment groups.

Given that RU486 inhibits androgen signaling, we began our studies on androgen-independent human prostate cancer cell line PC-3 tumors formed subcutanously in NOD.*scid *mice. As shown in Figure 1, both the monotherapy and combination therapy by IT administration of the Ad5IL-12 vector resulted in statistical significant attenuation of PC3 tumor growth compared to control treatments at the 8-week time point (two-tailed t-Test; p < 0.05). Ad5IL-12 vector treated mice had an approximate 5-fold greater reduction in PC3 tumor growth in comparison to the control DL70-3 adenovirus vector as well as to the PBS controls (668 ± 87 mm^3 ^versus 3163 ± 802 mm^3 ^and 3394 ± 707 mm^3^, respectively). These data were in agreement with our previous findings using this model system in which tumor regression was shown to be principally NK cell-dependent [8].

Here, addition of RU486 to the Ad5IL-12 vector led to even further tumor inhibition. Combination therapy resulted in mice with average tumor volumes of 298 ± 120 mm^3 ^at the 8 week time point, representing an additional 2.24-fold reduction in tumor mass when compared to the Ad5IL-12 vector treatment alone (p = 0.029) and a 6.70-fold difference against the RU486 treatment alone (p = 0.010). While the administration of RU486 alone did appear to slow tumor growth somewhat in comparison to the DL70-3 and PBS controls, this effect did not reach statistical significance over the time course analyzed (the tumor volume for RU486 treatment at 8 weeks averaged 1989 ± 307 mm^3^).

### Both Ad5IL-12 vector or RU486 treatment can attenuate the growth of human androgen-dependent LNCaP xenograft tumors

**Figure 2:**
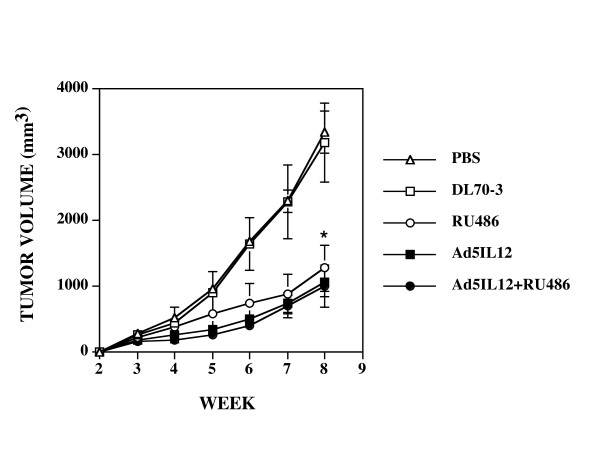
**Intratumor injection with Ad5IL-12 or 1 week treatment with RU486 attenuates the growth of human LNCaP tumors**. Xenograft tumors established in NOD.*scid *mice were treated at week 3 by IT injection with 50 μl of PBS containing 5 × 10^8 ^pfu of Ad5IL-12 (filled squares) or the control DL70-3 vector (empty squares) or PBS alone (empty circles). In addition, another set of mice were treated with Ad5IL-12 IT and given daily IP injections of RU486 for 7 days (black triangles). Data points are expressed as the mean ± SE. n = 8 for each data point. * indicates statistical significance of P < 0.05 for Ad5IL-12 + RU486 treatments alone compared to controls. Tumor volumes measured at 8 weeks were 3353 ± 532 mm^3 ^for PBS, 3197 ± 600 mm^3 ^for DL70-3, 1284 ± 350 for RU486, 1073 ± 226 mm^3 ^for Ad5IL-12 and 1015 ± 321 mm^3 ^for Ad5IL-12 + RU486 treatment groups.

We next investigated tumor treatments of androgen-dependent LNCaP xenograft tumors. As shown in Figure 2, statistical differences in tumor growth were demonstrated, with both Ad5IL-12 vector or RU486 treatment resulting in an approximate 3-fold reduction in tumor mass compared to controls (p < 0.05). Tumor volumes averaged 1073 ± 226 mm^3 ^in Ad5IL-12 vector treated mice in comparison to 3197 ± 600 mm^3 ^for DL70-3 vector and 3353 ± 532 mm^3 ^for PBS treatment. Unlike the limited effect seen for RU486 treatment against PC3 androgen-independent tumors, the mifepristione treatment regimen here alone was able to significantly attenuate LNCaP tumor growth. Also in contrast to the effect for combination therapy seen against PC3 tumors, the combined action of Ad5IL-12 and RU486 treatment did not produce a statistically significant better therapeutic effect against tumor than either treatment alone. At the 8 week time point, tumor volumes averaged 1284 mm^3 ^for RU486 treatment compared to 1073 mm^3 ^for Ad5IL-12 alone and 1015 mm^3 ^for the Ad5IL-12/RU486 combination treatment. For LNCaP tumors, the RU486 treatment regimen alone produced similar attenuation of tumor growth as that of Ad5IL-12 IT treatment. Our results support earlier findings for RU486 effects on LNCaP tumors but also indicate that the systemic delivery of RU486 (IP) can affect tissue-localized responses against an androgen-dependent tumor.

### Combination Ad5IL-12 + RU486 therapy in immune competent C57BL/6 mice produces significantly greater attenuation of TRAMP-C1 tumor growth than either treatment alone

**Figure 3:**
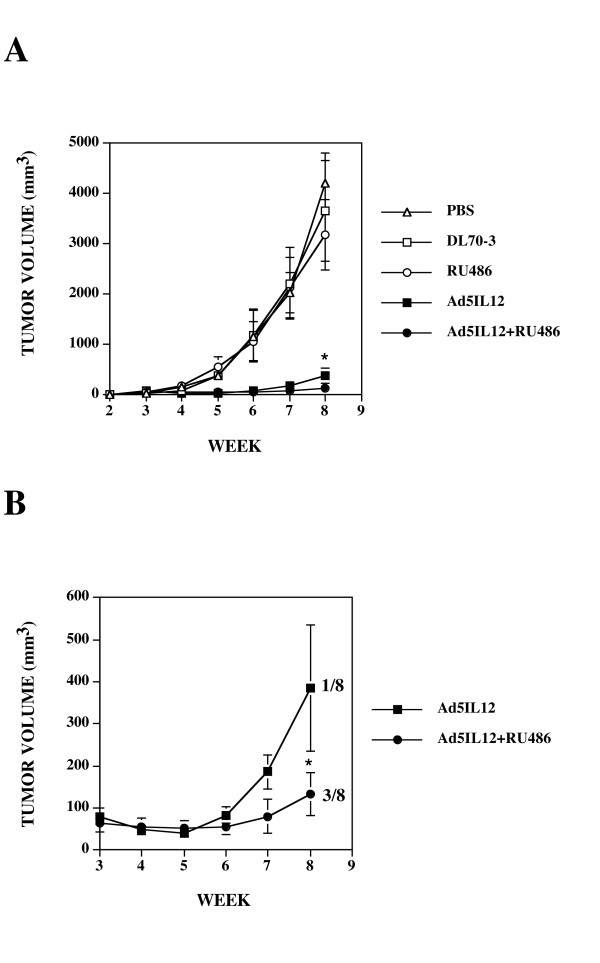
**Intratumoral injection with Ad5IL-12 vector and 1 week treatment with RU486 synergistically attenuates growth of TRAMP-C1 tumors**. (A) TRAMP-C1 tumors established in C57BL/6 mice were treated at week 3 following tumor cell inoculation by IT injection with 50 μl of PBS containing 5 × 10^8 ^pfu of Ad5IL-12 (filled squares) or control DL70-3 vector (empty squares) or PBS alone (empty circles). Data points are expressed as the mean ± SE. n = 8 for each data point. * indicates statistical significance of P < 0.01 for Ad5IL-12 compared to controls. (B) C57BL/6 mice treated with an intratumor injection of Ad5IL-12 (black squares), or given an additional daily IP injection with RU486 (black triangles) for 1 week were compared. * indicates statistical significance of P < 0.05 for Ad5IL-12 + RU486 compared to Ad5IL-12 alone. The ratio of cures per number of treated animals is indicated. Tumor volumes measured at 8 weeks were 4204 ± 604 mm^3 ^for PBS, 3661 ± 1049 mm^3 ^for DL70-3, 3194 ± 733 for RU486, 386 ± 77 mm^3 ^for Ad5IL-12 and 133 ± 53 mm^3 ^for Ad5IL-12 + RU486 treatment groups.

Because the use of NOD.*scid *mice bearing human xenograft prostate tumors does not model treatment effects on a fully intact immune system, we next set out to determine what impact combination therapy would have against established TRAMP-C1 tumors using immune competent C57Bl/6 mice. As shown in Figure 3A, treatment with a single IT injection of Ad5IL-12 vector caused significant reduction of TRAMP-C1 tumor growth (with much greater reductions) in comparison to control treatments (PBS, DL70-3 and RU486). Tumor volumes averaged 386 ± 77 mm^3 ^for Ad5IL-12 treatment in comparison to 4204 ± 604 mm^3 ^for PBS, 3661 ± 1049 mm^3 ^for DL70-3 and 3194 ± 733 mm^3 ^for RU486 treatment. In these immunocompetent mice, RU486 significantly augmented the effects of Ad5IL-12 vector treatment with an approximate 2.9-fold attenuation of tumor growth being evidenced in comparison to the Ad5IL-12 vector treatment alone (Figure 3B). Tumor volumes averaged 386 ± 77 mm^3 ^for Ad5IL-12 vector treated mice versus 133 ± 53 mm^3 ^in RU486 + Ad5IL-12 combination therapy. Statistically significant differences for effects on tumor growth (p < 0.05) were reached by the 8-week time point in comparison between the Ad5IL-12 vector alone versus combination Ad5IL-12+RU486 treatment indicating inclusion of RU486 improved therapeutic efficacy. Moreover, combination therapy produced a 24-fold greater attenuation of tumor growth in comparison to the RU486 treatment alone. This finding is striking considering here that RU486 treatment appeared to have no significant effect on TRAMP-C1 tumor growth alone. While no cures were produced by treatment from any control animals, 3 of 8 mice receiving the combination therapy had complete resolution of their tumors. As the TRAMP-C1 cells used in tumor formation were weaned off their androgen-dependency, these results suggest that RU486 treatment can better enhance the therapeutic effects by a proinflammatory cancer agent through immune-mediated mechanisms in an immune competent host.

### TSLN cells isolated following combination Ad5IL-12/RU486 treatment generate enhanced granzyme B levels against TRAMP-C1 tumor cell targets

**Figure 4:**
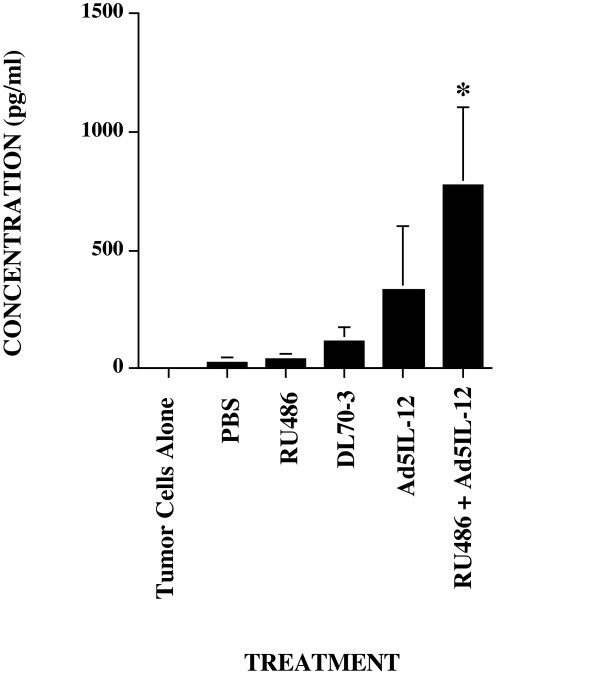
**Granzyme B production from cells is additionally enhanced following Ad5IL-12 and RU486 therapy**. Granzyme B levels were measured from isolated TSLN cells in TRAMP-C1 tumor bearing C57BL/6 mice following experimental treatments. Assays were performed in duplicate for each treated animal. Cumulative data from 2 independent experiments are shown using a total of n = 8 animals per each treatment group. * indicates statistical significance of P < 0.05 for Ad5IL-12 + RU486 treatments alone compared against all other treatment groups.

In tumor models involving subcutaneous flank implantation similar to the one used in these studies, the popliteal lymph node serves to provide lymphatic drainage and also contains the highest number of tumor-specific effector T cells [30]. To investigate possible mechanisms involved in the ability of RU486 to enhance efficacy of Ad5IL-12, we compared granzyme B levels produced from isolated popliteal lymph node cells (the TSLN) co-cultured for 24 hrs with irradiated TRAMP-C1 tumor cells as targets. Granzyme B is an important effector molecule of cell-mediated immunity correlating to effective tumor immune response [31] and measurement of its levels correlate well to total cellular cytotoxicity [32]. TSLN cells were isolated from individual animals with established TRAMP-C1 tumors following treatment. As shown in Figure 4, granzyme B levels in Ad5IL-12-treated mice were enhanced in comparison to the DL70-3, RU486 and PBS control treatment groups. Granzyme B levels averaged 337 pg/ml in Ad5IL-12 treated mice compared to 119 pg/ml for DL70-3, 32.8 pg/ml for RU486 or 5.5 pg/ml for PBS controls. An additional 2-fold increase in granzyme B production could be produced by (averaging 779 pg/ml) was found for combination RU486 + Ad5IL-12 vector treatment. Given the importance of the TSLN in tumor response [5], this additional increase in granzyme B production indicates that improved cytolytic activity can be facilitated by the addition of RU486 treatment to the Ad5IL-12 vector.

### Ly49C^+ ^NK cells are expanded by Ad5IL-12 therapy but cannot be further enhanced by combination therapy

**Figure 5:**
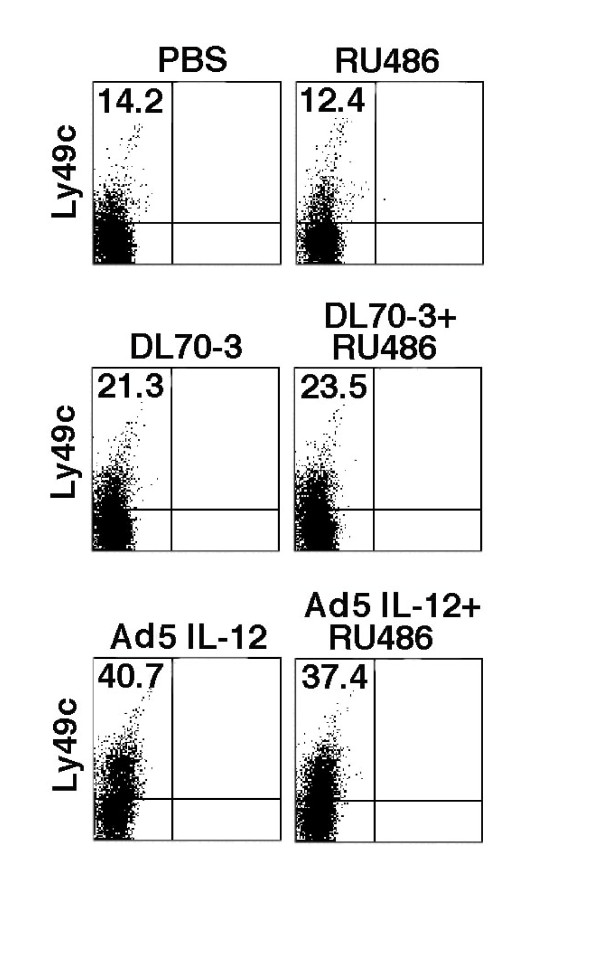
**NK cell populations in the TSLN are increased by Ad5IL-12 vector treatment**. TRAMP-C1 tumors in C57BL6 mice were treated with injection of PBS, DL70-3, or the Ad5IL-12 vector. Another set of mice corresponding to each of these treatment groups received an additional daily administration of RU486 IP for 1 week. At the end of treatment, TSLN were isolated and analyzed by flow cytometry for their content of Ly49C^+ ^NK cells. A representative dot plot is shown from one set of animals out of 3 separate experiments. Cumulative data from 3 flow cytometry analyses demonstrated Ly49C expression percentages averaged 7.95 ± 2.8 for PBS, 8.86 ± 2.7 for RU486, 11.94 ± 6.0 for DL70-3, 12.07 ± 4.7 for DL70-3 + RU486, 19.88 ± 9.9 for Ad5IL-12 and 21.33 ± 9.5 for Ad5IL-12 + RU486 treatment groups; n = 6. TSLN lymphocytes from two treated animals from each treatment were analyzed in each flow cytometry experiment.

We have previously reported that Ad5IL-12 therapy elicits antitumor effects through an NK cell-dependent response [8]. Accordingly, we sought to determine whether any enhancement in efficacy by the inclusion of RU486 was related to modulation of NK cell numbers at the level of the TSLN. To address this, flow cytometry was used to assess levels of Ly49C^**+ **^cells from TSLN isolated from TRAMP-C1 tumor bearing mice following the end of the treatment cycle. In Figure 5, a representative group of animals from one of the flow cytometry analyses is shown. In Ad5IL-12 treated mice, an approximate 2-fold increase in the percentage of Ly49C^+ ^NK cells was observed compared to DL70-3 controls (40.7% compared to 21.3%, respectively). Here, the addition of RU486 to Ad5IL-12 vector therapy did not increase the number of NK cell numbers elicited any greater than that of the Ad5IL-12 vector treatment alone. NK cell percentages for Ad5IL-12 + RU486 versus the Ad5IL-12 vector remained similar suggesting that NK cells may already be optimally expanded with Ad5IL-12 vector treatment. While the DL70-3 vector treatment resulted in an approximate 1.5 fold increase in the percentages NK cells found in the TSLN in comparison to the PBS control (21.3% compared to 14.2%, respectively), DL70-3 vector treatment had little overall impact on TRAMP-C1 tumor growth. Other factors in addition to the expansion of NK cells must account for the differences in the tumor killing produced between the Ad5IL-12 treatment groups and controls. The upregulation of FAS expression on NK cells has been shown to be mediated by IL-12 and could account for some of the enhanced tumor killing response [33].

### A trend towards decreases in regulatory T cells in the TSLN is found following combination therapy with Ad5IL-12 and RU486 in TRAMP-C1 tumor bearing C57Bl/6 mice

**Figure 6:**
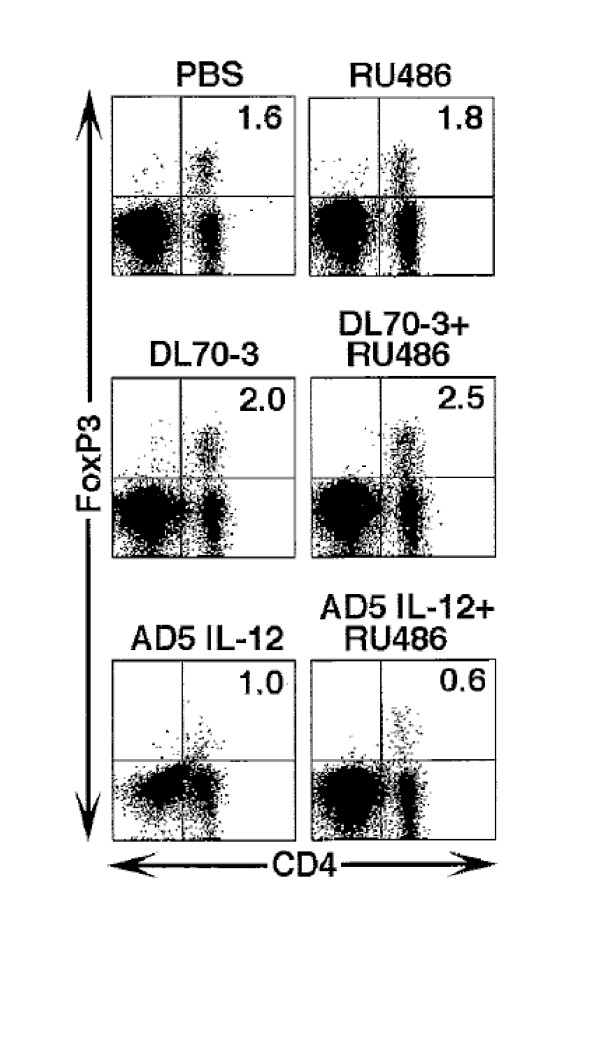
**Ad5IL-12 vector treatment of TRAMP-C1 tumors can reduce percentages of CD4/Foxp3 Tregs found in the TSLN**. C57BL6 mice were treated with injection of PBS, DL70-3, or the Ad5IL-12 vector while another set of mice corresponding to each of these treatment groups received an additional daily IP administration of RU486 for 1 week. At the end of this treatment, draining TSLN were isolated from individual animals and analyzed by flow cytometry for their content of CD4^+^/Foxp3^+ ^T cells. A representative dot plot is shown from one set of animals out of 3 separate experiments. Cumulative data from 3 flow cytometry analyses demonstrated CD4/FoxP3 expression percentages averaged 2.27 ± 0.2 for PBS, 2.12 ± 0.3 for RU486, 1.98 ± 0.2 for DL70-3, 1.98 ± 0.2 for DL70-3 + RU486, 1.75 ± 0.4 for Ad5IL-12 and 1.64 ± 0.4 for Ad5IL-12 + RU486 treatment groups; n = 6. TSLN lymphocytes from two treated animals from each treatment were analyzed in each flow cytometry experiment.

Regulatory T cells (Treg) have been implicated in the down regulation of tumor immunity in the TSLN [5]. As impairment of Treg function may be conferred by reductions in number, we evaluated the impact of combination therapy on the Treg compartment in the TSLN following completion of the experimental therapeutic regimen. In Figure 6, a representative group of animals from one of the flow cytometry analyses is shown. The percentage of CD4^+^Foxp3^+ ^T cells found in Ad5IL-12 treated mice were diminished in the TSLN in comparison to PBS and DL70-3 vector controls (1.0% versus 1.6% and 2.0%, respectively). An additional decrease in Treg content could found when RU486 was used in combination with the Ad5IL-12 vector versus the Ad5IL-12 vector treatment alone (0.6% versus 1.0%). Cumulative data of 6 animals in total from each treatment group revealed a trend towards lower Treg presence in the TSLN for the Ad5IL-12 (1.75 ± 0.35%) and Ad5IL-12 + RU486 (1.64 ± 0.36%) treatment groups in comparison to all the other treatment groups including the PBS (2.26 ± 0.27%) and DL70-3 (1.98% ± 0.18%) controls. Together, these data suggest that Treg cells may be influenced by cortisol in the TSLN and contribute in part to suppression of tumor immunity.

## Discussion

Mifepristone is a drug that has been previously approved for the termination of pregnancy and its capacity to act as an antagonist for the progesterone hormone receptor. However, it can also work as an antagonist for an additional array of hormone receptors including those of estrogen, testosterone and cortisol. Importantly, it has already been shown to have inhibitory effects on the growth of both ovarian and breast cancers in human clinical trials [34]. Because of the potential capacity to block cortisol signaling, we thought RU486 could act in addition as an immune modulatory agent and serve as a possible adjuvant in prostate cancer therapy. No reports for the effects of RU486 in combination with an immune stimulatory factor have yet been described to our knowledge. Interestingly, RU486 has been reported to impact cancer cachexia by blocking interaction of cortisol and induction of zinc-alpha2-glycoprotein (ZAG) expression in adipose tissue [35]. ZAG impacts the mobilization of fat stores and breakdown of body fat supporting another indication for the inclusion of RU486 in therapy. Thus, the use of RU486 in prostate cancer therapy could have effects on cachexia, androgen-dependent tumor growth and as an adjuvant in immune response activation. In this study, we have begun to address some of these considerations with regard to immune response and androgen-dependency.

Here, we have been able to demonstrate that the addition of RU486 (mifepristone) in combination with intratumor injection of Ad5IL-12 vector can enhance prostate cancer therapeutic efficacy versus that of vector therapy alone. The inclusion of RU486 may further enhance tumor immunity within the TSLN through a variety of factors. The addition of RU486 to Ad5IL-12 vector therapy enhanced tumor cytotoxicity as measured by granzyme B production against TRAMP-C1 tumor targets from isolated TSLN lymphocytes. In addition to its effect on cytotoxicity, inclusion of RU486 in Ad5IL-12 vector treatment appeared to lead to further subtle decreases in regulatory CD4 T cell populations to be recovered in the TSLN. Both of these effects would appear to be advantageous towards inducing better tumor immunity and protecting against the spread of tumor cells into the draining TSLN. While most of the anti-tumor effect is clearly the result of the proinflammatory response induced by the Ad5IL-12 vector, our results indicate that additional cortisol blockade by RU486 allows for and enhanced activation and perhaps prolongation of both innate and adaptive tumor immune responses.

It is clear that the effects observed on LNCaP tumors in this study were mediated by RU486 antagonistic interactions on androgen receptor. The use of mifepristone has previously been shown to inhibit the growth of LNCaP tumors formed in nude mice through interaction with the androgen receptor (AR) because of a unique AR-T877A mutation that is present in this cancer cell variant [36]. It is likely that RU486 may also affect other prostate cancer cell types as well, as double AR mutant metastatic prostate cancer cells containing substitutions of L701H and T877A have been found that use cortisol as a growth factor [37]. Thus, inclusion of RU486 could provide additional benefit in cancer therapy for some prostate tumors independent of its effect on immune response as an adjuvant we have found.

In what would appear to be a contra-indication for the use of RU486 in therapy, glucocorticoids are often prescribed to treat hormone refractory prostate cancers. However, the beneficial effects for this therapy are transient and are only found to help a small subset of patients (20 to 25% of all cases of disease) [38]. What could account for this small percentage of tumors found to be responsive to glucocorticoid treatment is the observation that the glucocorticoid receptor (GR) is lost in up to 85% of all prostate cancers during progression [39]. Thus the beneficial effect of glucocorticoid therapy may be limited to only a small subset of patients. From our results, it appears likely that the inclusion of RU486 (given during the therapeutic window of time) with an immunostimulatory agent could be beneficial in the treatment of most prostate cancer types but possibly affecting each through different mechanisms.

Previous studies have reported on the use of an Ad5IL-12 vector in experimental cancer therapy including prostate cancer with promising results including the ability to aide in the suppression of lung metastases [40,41]. The anti-tumor activities of IL-12 are known and include inducing NK cell activation and boosting the generation of antigen-specific immune response. The proinflammatory effect of IL-12 is more effective when applied in local tumor therapy versus systemic treatment due to its potential toxicity. The ability to deliver RU486 systemically and influence the local effects of IL-12 could limit some of the toxic effects of IL-12 and offer a general strategy to aid in the activity of other localized proinflammatory acting cancer agents.

Some studies have linked chronic inflammation to the initiation of prostate cancer and even further have suggested that Tregs can act in a protective manner against the generation of cancer [42]. We suggest this phenomenon is a consequence of timing as it is possible that chronic inflammation (and loss of control by Treg) could be deleterious and aid in cancer during early initiation events when genetic mutations can be acquired. It is likely that at later stages, when mutations have already been established, that removal of Treg and inducing inflammatory conditions in the tumor would be beneficial. In support of this idea, it has already been shown that antitumor immunity in cancer patients is enhanced by the elimination of Tregs [43] and an over-abundance of tissue CD4 Tregs leads to additional dysfunctions in antigen-specific CD8 T cell responses [44]. Finally, cancer patients with demonstrated increases of Treg in their circulation and an increased presence in their tumor tissues have poorer clinical outcomes [45,46].

Completion of a phase II clinical trial study using RU486 on castration resistant prostate cancers revealed limited benefit for this treatment [47]. Yet, this trial revealed good tolerance for mifepristone treatments especially in the elderly patient population studied with no incidences of clinical adrenal insufficiencies were reported. Similar low toxicity was witnessed for the repeated use of RU486 in ovarian and breast cancer studies indicating this drug is well tolerated in patients. The poor effects for RU486 in this previous prostate cancer study could reflect the selected patient sensitivity towards androgen alone. The ability of RU486 to influence immune response in conjunction with an immunostimulatory agent was not explored. We believe beneficial effect for this type of immune enhancement could be noticed in therapeutic application and should be tested. In our hands, RU486 treatment provided with the Ad5IL-12 pro-inflammatory agent was able to provide additional benefit for the control of human PC3 tumors (using only innate NK response) and TRAMP-C1 tumors (with a totally intact immune system and in the presence of Treg).

## Conclusion

Our results suggest that RU486 can be a clinically relevant agent for use as an adjuvant in pro-inflammatory cancer therapy and may help to override immunosuppressive conditions found within tumor microenvironments. We believe these results support the further development of combination therapy in cancer that include RU486 as an adjuvant and merits consideration for testing in human clinical trials.

## Competing interests

The authors declare that they have no competing interests.

## Authors' contributions

TB, CRG, AD and JG performed tumor inoculations and measurements. Granzyme B assays were performed by TB, AD and JG. Flow cytometry analysis was performed by TB, CRG and aided in analysis and production of figures by RH. TB, CRG and ES conceived and designed experiments. The Canadian collaborators FLG and JG provided adenovirus vectors. TB and CRG wrote the manuscript.

All authors have read and approved the manuscript.
